# Variable-stiffness prosthesis improves biomechanics of walking across speeds compared to a passive device

**DOI:** 10.1038/s41598-024-67230-3

**Published:** 2024-07-17

**Authors:** Emily Rogers-Bradley, Seong Ho Yeon, Christian Landis, Duncan R. C. Lee, Hugh M. Herr

**Affiliations:** 1 K. Lisa Yang Center for Bionics, Massachusetts Institute of Technology, Cambridge, 02139 USA; 2 Department of Mechanical Engineering, Massachusetts Institute of Technology, Cambridge, 02139 USA; 3 Media Lab, Massachusetts Institute of Technology, Cambridge, 02142 USA; 4 Department of Mechanical and Manufacturing Engineering, University of Calgary, Calgary, T2N 1N4 Canada

**Keywords:** Biomedical engineering, Translational research

## Abstract

Ankle push-off power plays an important role in healthy walking, contributing to center-of-mass acceleration, swing leg dynamics, and accounting for 45% of total leg power. The majority of existing passive energy storage and return prostheses for people with below-knee (transtibial) amputation are stiffer than the biological ankle, particularly at slower walking speeds. Additionally, passive devices provide insufficient levels of energy return and push-off power, negatively impacting biomechanics of gait. Here, we present a clinical study evaluating the kinematics and kinetics of walking with a microprocessor-controlled, variable-stiffness ankle-foot prosthesis (945 g) compared to a standard low-mass passive prosthesis (Ottobock Taleo, 463 g) with 7 study participants having unilateral transtibial amputation. By modulating prosthesis stiffness under computer control across walking speeds, we demonstrate that there exists a stiffness that increases prosthetic-side energy return, peak power, and center-of-mass push-off work, and decreases contralateral limb peak ground reaction force compared to the standard passive prosthesis across all evaluated walking speeds. We demonstrate a significant increase in center-of-mass push-off work of 26.1%, 26.2%, 29.6% and 29.9% at 0.75 m/s, 1.0 m/s, 1.25 m/s, and 1.5 m/s, respectively, and a significant decrease in contralateral limb ground reaction force of 3.1%, 3.9%, and 3.2% at 1.0 m/s, 1.25 m/s, and 1.5 m/s, respectively. This study demonstrates the potential for a quasi-passive microprocessor-controlled variable-stiffness prosthesis to increase push-off power and energy return during gait at a range of walking speeds compared to a passive device of a fixed stiffness.

## Introduction

Ankle push-off power—the positive power generated by the ankle during the late-stance phase of human walking—is critical to healthy gait, generating approximately 45% of total leg power^1^, contributing to forward acceleration of the center of mass (CoM) of the body^2,3^, and aiding in swing phase initiation^2,3^. The most commonly used prostheses for people with TTA, passive energy storage and return (ESR) prostheses, provide lower than physiological levels of push-off power from the prosthetic side^4,5^. In healthy walking it has been demonstrated that reduced ankle push-off power is correlated with increased metabolic energy requirements^6,7^ and increased mechanical energy loss during step transitions^7^. For people with unilateral transtibial (below-knee) amputation (TTA), there has been an identified link between lower levels of ankle push-off power and increased contralateral limb loading, measured by vertical ground reaction force (GRF) and knee external adduction moment (EAM)^4^. This increase in contralateral limb loading potentially contributes to the knee osteoarthritis incidence that is 17x higher than that of the general population^8^, and rates of knee pain twice as high as in people without amputation^9^.

A second category of device—powered prostheses—provide net positive work to the wearer and physiological levels of mechanical push-off power. It has been demonstrated that a powered ankle-foot prosthesis that provides net positive work to the user during stance, and provides higher levels of push-off power than ESR prostheses, can decrease knee EAM and vertical GRF on the contralateral limb, further demonstrating the correlation between prosthesis push-off power and contralateral limb loading^10^. Increasing prosthesis push-off power with powered protheses has been shown to reduce metabolic energy expenditure during walking^11,12^, although prosthetic emulator studies show mixed results^13,14^. Currently, the Empower by Ottobock is the only commercially-available powered ankle-foot prosthesis on the market^15^. Despite its clinical advantages, it is estimated that only 5% or less of the population with below-knee amputation use the Empower, primarily due to the lack of reimbursement by insurance^16,17^. Given that it provides net positive work and biomimetic levels of mechanical power at fast walking speeds, the Empower is necessarily heavier, larger, and more expensive compared to ESR prostheses^15^.

A third category of ankle-foot prosthetic design is quasi-passive. This type of prosthesis can offer microprocessor-controlled joint position control during the swing phase of gait^18–20^, adjust neutral angle during swing^21^, adjust ankle or forefoot stiffness^22–25^, or recycle heel strike energy^26^. Although microprocessor-controlled with a power source, quasi-passive ankle-foot prostheses are not designed to provide net positive work during the stance phase, and are therefore lower in weight, size, and cost compared to a fully powered prosthesis. A quasi-passive controlled energy-storage prosthesis has been used to demonstrate that increasing energy return from the prosthesis increases prosthetic side CoM push-off work and decreases intact limb collision work^27^ and EAM^4^.

In the biological ankle, joint stiffness adjustment is an important physiological function. During the controlled dorsiflexion phase of stance, the ankle behaves largely as a spring, with a first-order relationship between ankle moment and angle^28^. Prior work has demonstrated that ankle quasi-stiffness—the slope of the torque-angle relationship of the joint during dorsiflexion^29^—increases with increasing walking speed^28,30,31^, proportionally to load carriage^32^, with increasing ground compliance^33^, and to increase stability during standing^34^. During running, humans vary total leg stiffness to adapt to ground surfaces of varying compliance to modulate CoM oscillations^35^. In hopping, it has been demonstrated that the primary mechanism for adjusting total leg stiffness is modulation of ankle quasi-stiffness^36^. Commercial ESR prostheses have a passive spring stiffness that is significantly higher than the physiological ankle, particularly at slower walking speeds and across slopes and stairs^30,37^.

Variable-stiffness prostheses have previously been developed that adjust forefoot bending stiffness^22^ or ankle stiffness^23–25^ through adjustment of effective beam length of a cantilever beam^23,25^, length of overhung beam^22^, or a clutchable pneumatic cylinder^24^. The development of these quasi-passive devices has enabled valuable insight into the role of changing stiffness on the biomechanics of walking. Variable-stiffness prosthetic ankle devices have demonstrated increased dorsiflexion range of motion with a decrease in ankle stiffness^23,38^ and increased energy storage for lower stiffnesses^23^. Experiments on preferred stiffness and perception of prosthetic ankle stiffness change by users^39,40^, as well as the stiffness preferences of prescribing prosthetists^41^ has shown a preference for lower stiffnesses at slower walking speeds by users^40^, and higher stiffnesses preferred by prosthetists than by the users at preferred walking speeds^41^.

While forefoot stiffness differs from ankle stiffness, prior work analyzing the effects of foot stiffness during walking provides important insight for work on ankle stiffness. Prior experiments include a pilot study with three study participants at a fixed walking speed, demonstrating that decreasing forefoot bending stiffness increases energy storage and return and increases peak prosthetic power^22^. Further studies with the variable-stiffness foot device estimated the sensitivity of energy return, prosthetic power, and prosthetic-side kinematics to forefoot stiffness at a single walking speed, demonstrating increased energy storage, peak prosthetic power, and push-off work with lower levels of forefoot stiffness^42^.

The role of stiffness on gait has been further explored through passive devices that are manually adjusted for stiffness^43–45^. A study at one walking speed with varying keel stiffness of an ESR device demonstrated an increase in prosthetic side and contralateral side range of motion with decreasing stiffness, and a decrease in intact limb first peak of GRF^43^. A decrease in metabolic cost with decreasing ankle stiffness was demonstrated with an experimental passive adjustable stiffness ankle^45^, although the opposite conclusion was drawn from a prosthetic emulator study during experiments with load carriage^46^. Another study analyzed biomechanics at a range of walking speeds, demonstrating that decreasing prosthetic forefoot stiffness increased prosthetic side CoM push-off work^44^. Additional studies have analyzed the effect of powered prostheses with adjustable quasi-stiffness to mimic physiological torque-angle characteristics of the ankle and knee^47^ and to improve walking stability^48^. As powered prostheses are capable of providing positive joint work to the user during stance, as well as their much higher device mass, the present paper focuses largely on comparison with passive and quasi-passive devices.

There has yet to be a microprocessor-controlled variable-stiffness ankle-foot prosthesis that demonstrates an improvement in full body biomechanics across a range of walking speeds. While providing important biomechanical insights, the majority of prior studies analyze biomechanics at a single walking speed^22,38,39,42^, do not evaluate whole-body mechanics^40^, or evaluate effects of forefoot stiffness only^44^. Furthermore, prior studies do not provide comparison with standard passive devices, preventing conclusions regarding practical benefits of such variable-stiffness devices or their relative potential impacts across walking speeds compared to lower-mass passive devices operating at fixed stiffnesses. Here, we present a clinical gait study that evaluates a variable-stiffness ankle-foot prosthesis that utilizes computer-controllable, parallel-sliding composite leaf springs^49^. The multi-leaf spring architecture presents technical advantages over existing variable-stiffness designs such as reduced maximum bending stress for a given deflection and a high strength to mass ratio of carbon fiber composite^50^. Across a range of walking speeds, we hypothesize that with an autonomous microprocessor-controlled variable-stiffness prosthesis, despite the inherent increase in mass compared to a passive device and the mechanical energy loss due to sliding contact forces at the spring interface, we can demonstrate an increase in energy return, prosthetic peak power, and center of mass push off work compared to a standard low-mass passive prosthesis operating at a fixed stiffness. This device has the potential to improve the biomechanics of walking across a range of walking speeds. This biomimetic functionality of variable ankle stiffness aims to emulate the variable joint compliance of the biological ankle-foot complex exhibited across walking speeds and standing^28,30,51–54^. We also anticipate that through this increase in stance-phase prosthetic peak power we will see a decrease in contralateral limb loading and knee EAM, potentially decreasing risk factors for knee osteoarthritis development. This quasi-passive method of increasing peak prosthetic power aims to harness the $\theta ^2$ term in energy storage (Eq. 1) in order to increase energy storage in early stance and increase energy return and therefore peak power during step transitions.

1 $$\begin{aligned} E=\int \tau d\theta = k\theta ^2 \end{aligned}$$

The approach for increasing energy storage and return using the study’s variable-stiffness prosthesis is illustrated in Eq. 1. For stiffness *k*, we have an angular displacement ($\theta$) that occurs for a given torque ($\tau$). The energy stored in this spring is given by Eq. 1. For a decrease in stiffness, a larger angular displacement ($\theta$) occurs for the same applied torque ($\tau$). Larger energy storage and return will correspond to greater peak power, and thus by tuning prosthetic stiffness to biological stiffness levels across walking speeds, we hypothesize that energy storage and peak power will be increased. In other words, a passive prosthesis with a fixed stiffness is overly stiff for the majority of walking speeds, and therefore is wasting elastic potential energy storage and return.

**Figure 1:**
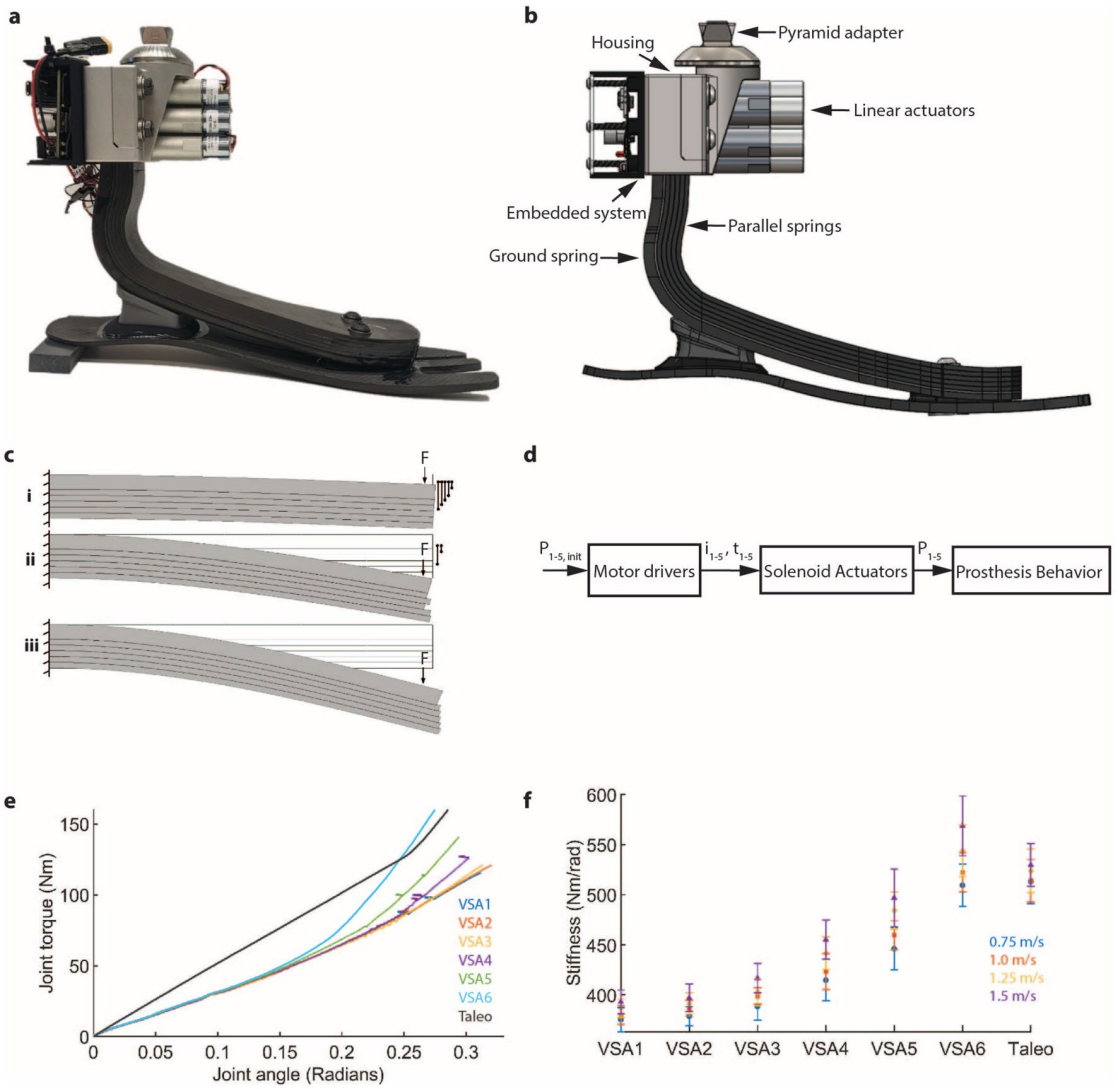
System overview. (**a**) Variable-stiffness ankle-foot prosthesis, (**b**) Rendering of the device showing ground spring, lockable parallel springs, solenoid driven linear actuators, structural housing, embedded system, and pyramid adapter, (**c**) Diagram demonstrating stiffness change mechanism utilizing independently-controlled locking parallel sliding leaf springs. Displacement of springs is represented for the same force (F) applied in each of: the stiffest configuration (**c-i**), one of the 30 distinct intermediate stiffness states (**c-ii**), and the lowest stiffness configuration (**c-iii**), (**d**) Control system block diagram demonstrating the implemented open-loop control system used in the presented study, (**e**) Torque-angle profile for variable stiffness ankle at the 6 evaluated stiffnesses and the control Taleo device (**f**) Effective mean stiffness of variable stiffness ankle and Taleo during biomechanical evaluation.

This paper presents clinical results from a study with 7 participants with unilateral transtibial amputation. We show that a microprocessor-controlled variable-stiffness ankle-foot prosthesis increases stance phase energy storage, peak power, and decreases contralateral limb loading compared to a passive ESR prosthesis. The experimental prosthesis (Fig.  1) weighs 945 g, has a computer adjustable nominal stiffness range of 375–569 Nm/rad, and has a build height of 162 mm^49^. During study experiments, subjects walked on an instrumented treadmill at the speeds of 0.75 m/s, 1.0 m/s, 1.25 m/s, and 1.5 m/s with the variable-stiffness prosthesis at 6 representative stiffnesses and a commercial passive ESR prosthesis (Ottobock Taleo) with a device mass of 463 g while kinetic and kinematic data were recorded. This paper presents results demonstrating that the variable-stiffness prosthesis increases peak joint power, energy return, and CoM push-off power compared to the passive low-mass Taleo prosthesis having a fixed stiffness. For the contralateral limb, the variable-stiffness prosthesis decreases CoM collision power and early stance peak vertical ground reaction force. The presented variable-stiffness prosthesis has the potential to expand access to high performance prosthetic technology by creating a device that has a lower mass, electrical power, and cost compared to a fully powered device.

## Results

**Figure 2:**
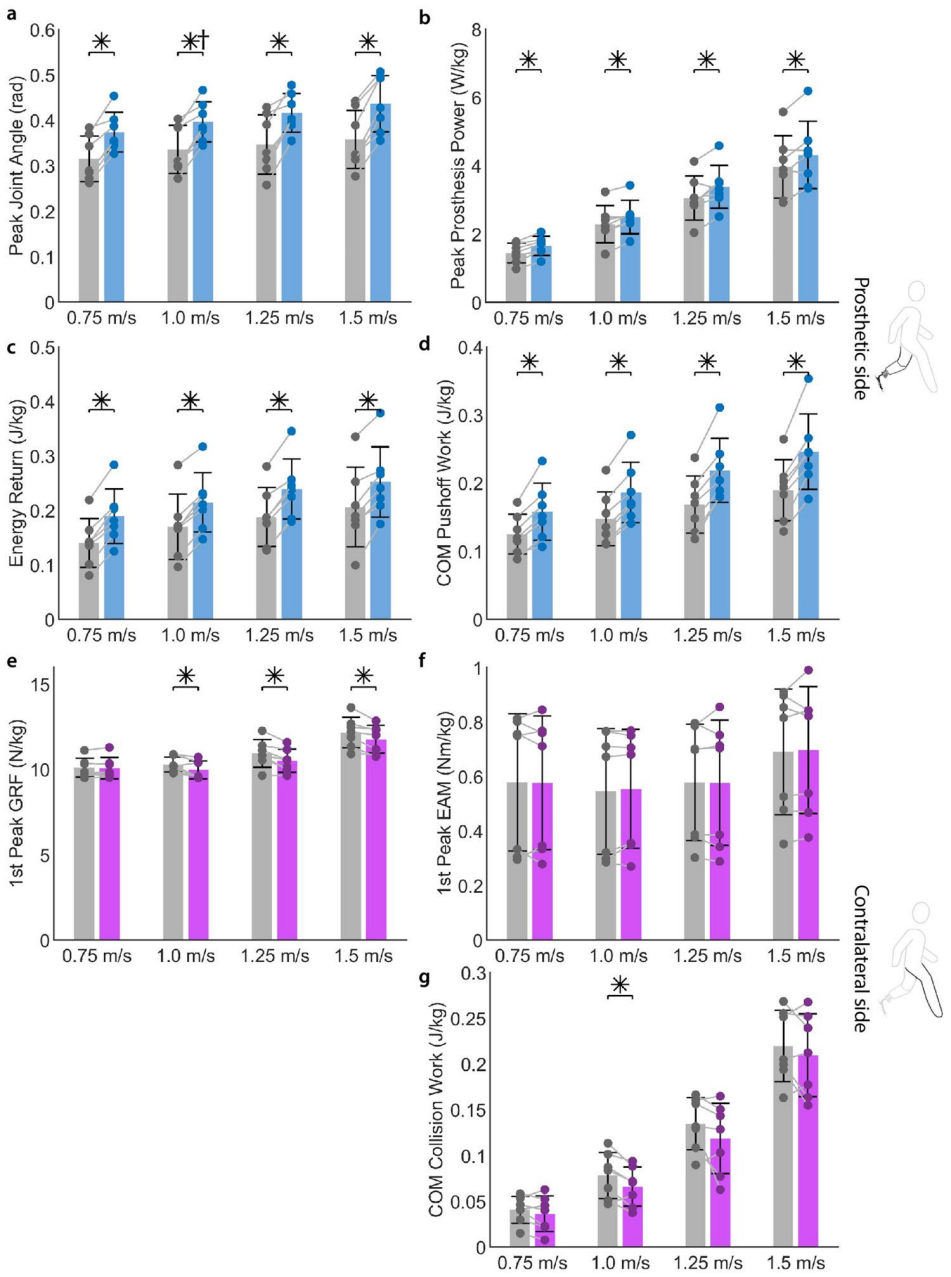
Mean results. Mean values of metrics of interest between participants for prosthetic side **(a)** peak joint angle, **(b)** peak prosthetic power, **(c)** energy return, **(d)** CoM push-off work, and contralateral side **(e)** peak GRF, **(f)** peak EAM, and **(g)** CoM collision work. Gray represents Taleo and variable stiffness ankle is represented by blue or purple. * indicates statistical significance ($\hbox {N}=7$, $\hbox {p}<0.05$). Paired t-test performed on all samples excluding condition marked by †, for which a paired Wilcoxon signed rank test was performed.

**Figure 3:**
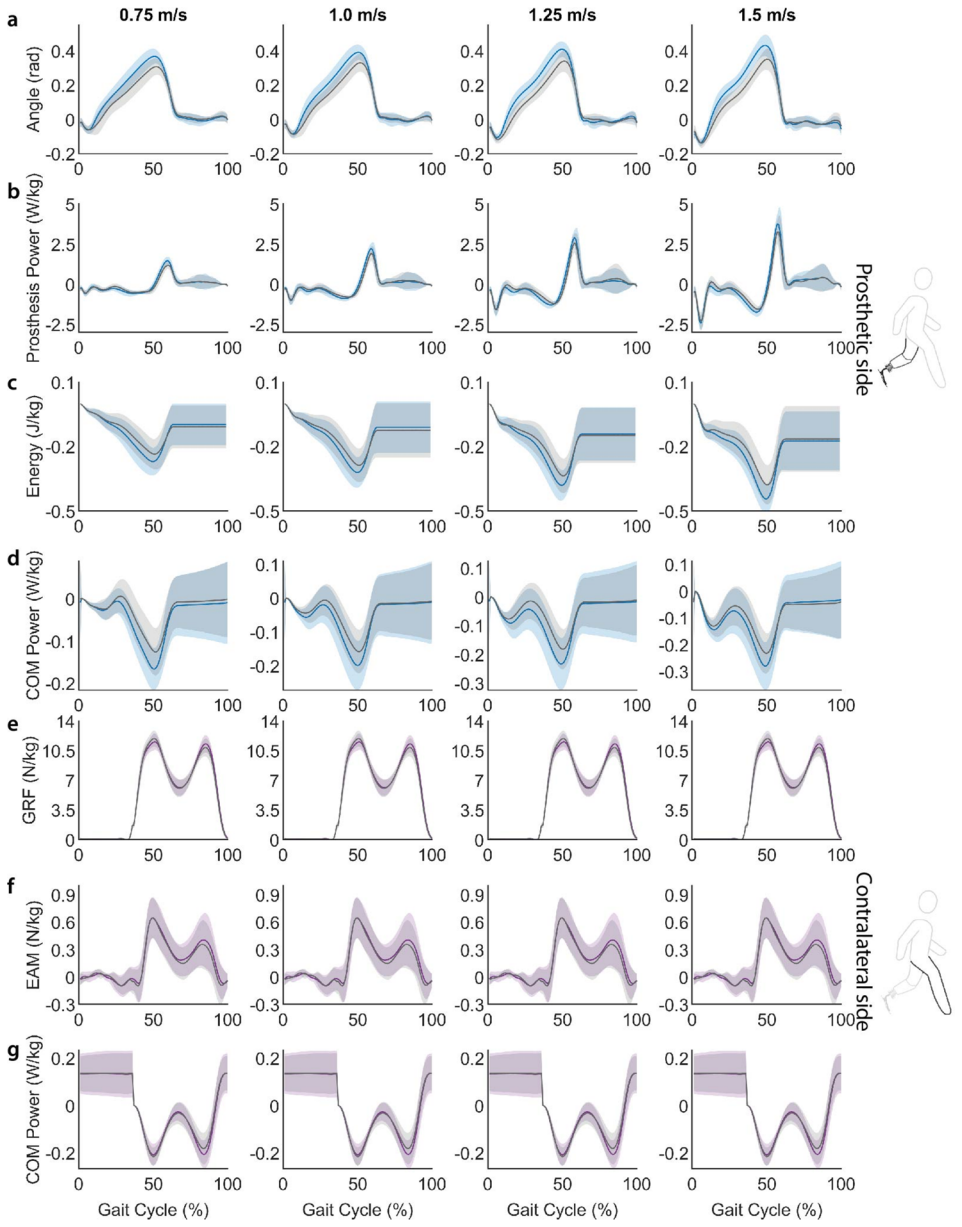
Results across gait cycle. Average values across gait cycle between all participants for prosthetic side (**a**) joint angle, (**b**) power, (**c**) energy, (**d**) CoM push-off power, and contralateral side (**e**) GRF, (**f**) EAM, and (**g**) CoM collision power. Gray represents the taleo conditions, with the VSA conditions shown in blue (prosthetic side) or purple (contralateral side). Solid lines indicate average values across the 7 participants, and shaded regions are $\pm 1$ standard deviation.

By modulating prosthetic ankle stiffness under computer control with the variable-stiffness ankle-foot (VSA) prosthesis, prosthetic side energy return increased and contralateral side loading decreased. On the prosthetic side, the VSA prosthesis demonstrated an increase in peak joint angle, peak power, energy storage and return, and CoM push-off work compared to walking with a passive prosthesis. On the contralateral limb we saw a decrease in early stance peak ground reaction forces compared to a passive device. Figures 2, 3, and Table 1 summarize these results. Table 2 presents the VSA stiffness for the condition that maximizes CoM push-off work for each participant at each evaluated walking speed.

**Table 1:** Results. VSA compared to passive Taleo.

Variable	Prosthesis	0.75 m/s	1.0 m/s	1.25 m/s	1.5 m/s
Peak ankle angle (rad)	Taleo	$0.32 \pm 0.05$	$0.34 \pm 0.05$	$0.35 \pm 0.07$	$0.36 \pm 0.06$
	VSA	$0.37 \pm 0.04$	$0.40 \pm 0.04$	$0.42 \pm 0.04$	$0.44 \pm 0.06$
	% change	$+18.7\%$	$+18.0\%$	$+20.1\%$	$+22.0\%$
	Significance	$\hbox {p}<0.001$*	$\hbox {p}=0.016$* †	$\hbox {p}=0.002$*	$\hbox {p}<0.001$*
	Effect size	1.09	1.08	1.10	1.08
Peak prosthesis power (W/kg)	Taleo	$1.44 \pm 0.29$	$2.29 \pm 0.55$	$3.06 \pm 0.64$	$3.97 \pm 0.91$
	VSA	$1.66 \pm 0.28$	$2.50 \pm 0.49$	$3.39 \pm 0.63$	$4.32 \pm 0.99$
	% change	$+15.0 \%$	$+9.0\%$	$+10.7\%$	$+8.8\%$
	Significance	$\hbox {p}<0.001$*	$\hbox {p}=0.006$*	$\hbox {p}=0.026$*	$\hbox {p}=0.022$*
	Effect size	0.66	0.35	0.45	0.32
Prosthesis energy return (J/kg)	Taleo	$0.14 \pm 0.04$	$0.17 \pm 0.06$	$0.19 \pm 0.05$	$0.21 \pm 0.07$
	VSA	$0.19 \pm 0.05$	$0.21 \pm 0.05$	$0.24 \pm 0.06$	$0.25 \pm 0.07$
	% change	$+35.0\%$	$+26.3\%$	$+27.4\%$	$+22.5\%$
	Significance	$\hbox {p}<0.001$*	$\hbox {p}<0.001$*	$\hbox {p}<0.001$*	$\hbox {p}<0.001$*
	Effect size	0.90	0.68	0.82	0.58
1st peak EAM (Nm/kg)	Taleo	$0.58 \pm 0.25$	$0.55 \pm 0.23$	$0.58 \pm 0.21$	$0.69 \pm 0.23$
	VSA	$0.58 \pm 0.25$	$0.55 \pm 0.22$	$0.58 \pm 0.23$	$0.70 \pm 0.23$
	% change	$-0.3\%$	$+1.6\%$	$-0.2\%$	$+1.0\%$
	Significance	$\hbox {p}=0.93$	$\hbox {p}=0.40$	$\hbox {p}=0.94$	$\hbox {p}=0.70$
	Effect size	0.01	0.03	0.00	0.03
1st peak GRF (N/kg)	Taleo	$10.11 \pm 0.55$	$10.30 \pm 0.43$	$10.96 \pm 0.80$	$12.17 \pm 0.90$
	VSA	$10.08 \pm 0.63$	$9.98 \pm 0.53$	$10.53 \pm 0.68$	$11.78 \pm 0.82$
	% change	$-0.3\%$	$-3.1 \%$	$-3.9 \%$	$-3.2\%$
	Significance	$\hbox {p}=0.6782$	$\hbox {p}=0.0378$*	$\hbox {p}=0.0084$*	$\hbox {p}=0.0127$*
	Effect size	0.04	0.57	0.50	0.40
COM collision work (J/kg)	Taleo	$0.041 \pm 0.015$	$0.079 \pm 0.025$	$0.135 \pm 0.029$	$0.220 \pm 0.039$
	VSA	$0.036 \pm 0.019$	$0.066 \pm 0.021$	$0.119 \pm 0.039$	$0.210 \pm 0.045$
	% change	$-10.8\%$	$-15.8\%$	$-12.0\%$	$-4.5\%$
	Significance	$\hbox {p}=0.2383$	$\hbox {p}=0.028$*	$\hbox {p}=0.1612$	$\hbox {p}=0.3179$
	Effect size	0.22	0.46	0.41	0.20
COM push-off work (J/kg)	Taleo	$0.125 \pm 0.029$	$0.148 \pm 0.039$	$0.169 \pm 0.042$	$0.190 \pm 0.045$
	VSA	$0.158 \pm 0.042$	$0.189 \pm 0.044$	$0.219 \pm 0.047$	$0.246 \pm 0.055$
	% change	$+26.1 \%$	$+26.2\%$	$+29.6\%$	$+29.9\%$
	Significance	$\hbox {p}=0.001$*	$\hbox {p}<0.001$*	$\hbox {p}<0.001$*	$\hbox {p}<0.001$*
	Effect size	0.79	0.80	0.98	0.98

**Table 2:** Selected stiffness values.

Subject	0.75 m/s (Nm/rad)	1.0 m/s (Nm/rad)	1.25 m/s (Nm/rad)	1.5 m/s (Nm/rad)
1	374.6	377.3	382.6	399.0
2	360.3	365.5	386.1	408.8
3	381.7	404.0	390.2	400.0
4	372.1	382.6	395.4	402.1
5	372.5	441.0	456.6	473.8
6	397.4	395.6	408.6	421.5
7	368.9	385.2	398.4	483.4

### Prosthetic side joint angle, power, energy return, and push-off work increased

There was an increase in maximum joint angle of the prosthesis across speeds for the VSA compared to the passive device. Mean peak joint angle for the VSA stiffness that maximizes CoM push-off work (blue) is greater than the passive ESR device (gray) across the 7 subjects (Fig. 2A). Joint angle across the gait cycle is greater for the VSA compared to the passive device at the four evaluated speeds (Fig. 3A). Peak dorsiflexion angle increased significantly ($\hbox {p}<0.05$) for the variable-stiffness ankle compared to the passive device at 0.75 m/s (paired t-test, $\hbox {p}<0.001$), 1.0 m/s (Wilcox paired signed rank, $\hbox {p}=0.016$), 1.25 m/s (paired t-test, $\hbox {p}=0.002$), and 1.5 m/s (paired t-test, $\hbox {p}<0.001$). The observed increase in peak dorsiflexion angle with the variable-stiffness prosthesis is 18.7%, 18.0%, 20.1%, and 22.0% at 0.75 m/s, 1.0 m/s, 1.25 m/s, and 1.5 m/s, respectively.

Mean peak power from the prosthesis is greater for the selected VSA stiffness compared to the passive device, with the greatest percentage difference in peak power at the slowest evaluated walking speed (Fig. 2B). The VSA selected stiffness compared to the passive device demonstrated a significant (paired t-test, $\hbox {p}<0.05$) increase in peak power at 0.75 m/s ($\hbox {p}<0.001$), 1.0 m/s ($\hbox {p}=0.006$), 1.25 m/s ($\hbox {p}=0.026$), and 1.5 m/s ($\hbox {p}=0.022$). The observed increase in peak power was 15.0% at 0.75 m/s, 9.0% at 1.0 m/s, 10.7% at 1.25 m/s, and 8.8% at 1.5 m/s. We observe an increase in peak negative power in mid-stance followed by an increase in positive power in late stance with the VSA compared to the passive device (Fig. 3B).

We observed a significant (paired t-test, $\hbox {p}<0.05$) increase in energy return at 0.75 m/s ($\hbox {p}<0.001$), 1.0 m/s ($\hbox {p}<0.001$), 1.25 m/s ($\hbox {p}<0.001$), and 1.5 m/s ($\hbox {p}<0.001$) for the VSA compared to the passive device. The VSA showed an increase in energy return of 35.0% at 0.75 m/s, 26.3% at 1.0 m/s, 27.4% at 1.25 m/s, and 22.5% at 1.5 m/s. Mean energy return averaged across the 7 participants form the VSA prosthesis is greater than the passive ESR device, with a greater difference in energy return at slower waling speeds (Fig. 2C). Energy is stored in the prosthesis from early to mid stance and returned during push-off in late stance (Fig. 3C).

CoM push-off work from the prosthesis side increased significantly (paired t-test, $\hbox {p}<0.05$) while walking with the VSA compared to the passive device at 0.75 m/s ($\hbox {p}=0.001$), 1.0 m/s ($\hbox {p}<0.001$), 1.25 m/s ($\hbox {p}<0.001$), and 1.5 m/s ($\hbox {p}<0.001$). We observed an increase in total push-off work equal to 26.1%, 26.2%, 29.6%, and 29.9% at 0.75 m/s m/s, 1.0 m/s, 1.25 m/s, and 1.5 m/s, respectively (Fig. 2D). Across the gait cycle, we observe a larger amount of negative CoM power in mid-stance and a greater peak CoM power in late stance with the VSA compared to the passive device (Fig. 3D).

### Contralateral side vertical ground reaction force decreased

For three of the evaluated walking speeds, there is a decrease in contralateral side vertical ground reaction force (GRF) for the selected VSA compared to the passive device (Fig. 2E). We observed a significant (paired t-test, $\hbox {p}<0.05$) decrease in the 1st peak of vertical GRF of the contralateral limb during step transitions at 1.0 m/s ($\hbox {p}=0.038$), 1.25 m/s ($\hbox {p}=0.008$), and 1.5 m/s ($\hbox {p}=0.013$). The 1st peak of the contralateral ground reaction force was decreased by 3.1% at 1.0 m/s, 3.9% at 1.25 m/s and 3.2% at 1.5 m/s. Across the gait cycle, there is a lower 1st peak of vertical GRF of the contralateral limb for the VSA compared to the passive ESR device averaged across the 7 subjects (Fig. 3E).

Compared to walking with the passive prosthesis, the variable-stiffness prosthesis did not show a significant difference in EAM about the knee at any speed. The mean 1st peak of contralateral side EAM is similar for the VSA compared to the passive ESR device (Fig. 2F). Similarly, across gait cycle contralateral EAM is similar for the VSA prosthesis compared to the passive device at each of the four evaluated speeds (Fig. 3F).

There was a notable decrease in CoM collision work of the contralateral limb for one of the four evaluated walking speeds (Fig. 2G). CoM collision work was decreased significantly (paired t-test, $\hbox {p}<0.05$) at 1.0 m/s ($\hbox {p}=0.028$). There was a decrease of 15.8% at 1.0 m/s. Across gait cycle, contralateral CoM power demonstrates a lower amount of negative power during heel strike for the 1.0 m/s condition for the VSA prosthesis compared to the passive device (Fig. 3G).

### Selected prosthetic stiffness

**Figure 4:**
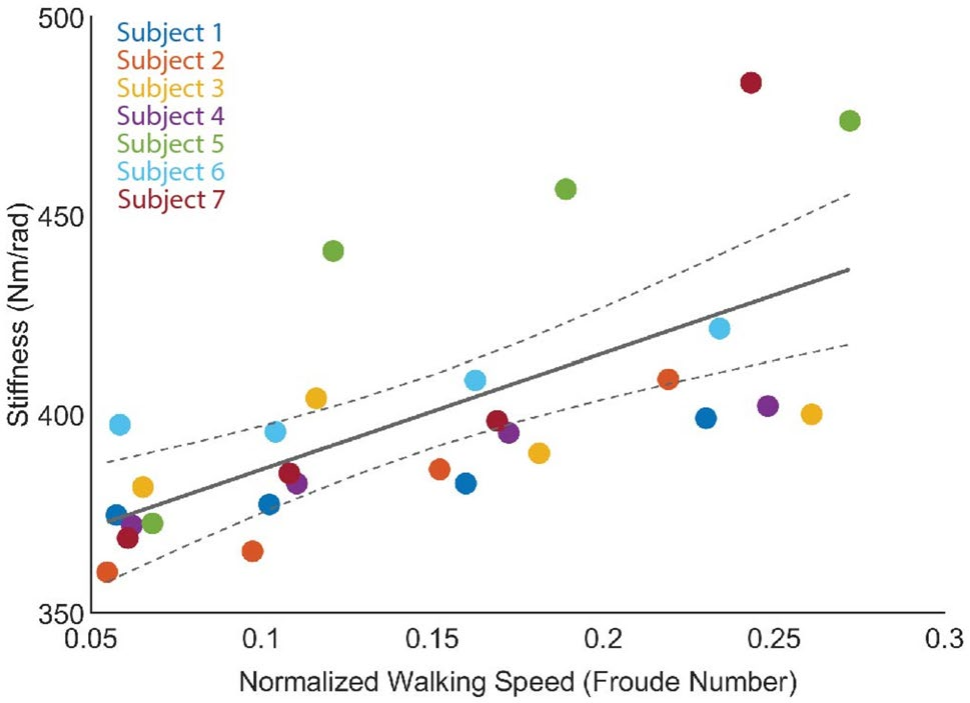
Correlation between normalized walking velocity and VSA stiffness that maximizes CoM push-off work. Data from each subject are plotted, demonstrating a significant positive correlation between Froude number ($v_{walking}^2/gl_{leg}$) and prosthetic ankle stiffness. The solid line represents the first-order linear regression between Froude number and stiffness. Dashed lines represent the 95% confidence interval of this model.

There is a positive linear relationship between walking speed (as represented by the dimensionless Froude number (Eq. 9)) and the selected prosthetic ankle stiffness that maximizes CoM push-off work (Fig. 4). The estimated VSA stiffness for the condition that maximized CoM push-off work for each participant is summarized in Table 2. The relationship between selected prosthetic stiffness and the dimensionless Froude number (Eq. 2) has a coefficient of determination ($R^2$) of 0.45, the RMS error is 23.5, and the p-value compared to a constant model is $\hbox {p}=0.0001$.

2 $$\begin{aligned} K_{VSA}=292.1Fr + 356.9 \end{aligned}$$

### Device cycle life

Cycle life testing demonstrated device durability of over 2x$10^6$ cycles without structural failure. During testing, Heel reference ankle moment (RAM) remained consistent within 10% of the initial heel RAM (Figure S8). Toe RAM decreased over the course of testing by 21 Nm (16% of initial toe RAM) (Figure S8).

## Discussion

This study demonstrates the ability to increase prosthetic energy return and peak power, and decrease contralateral limb loading across a range of walking speeds through the use of a microprocessor-controlled, variable-stiffness prosthesis. This work demonstrates that despite the inherent technical trade-offs of a quasi-passive system compared to a passive device such as increased device mass, spring hysteresis energetic losses, and mechatronic complexity, increases in peak prosthesis power, energy return, and CoM push-off work are measured across all evaluated walking speeds.

The presented clinical results demonstrate a significant increase in prosthetic ankle range of motion, peak ankle power, stored and returned energy, and COM push-off work. Additionally, we demonstrate a significant decrease in contralateral GRF at 3 of the 4 evaluated walking speeds, and decrease in contralateral CoM collision work at one evaluated speed. The selected prosthetic ankle stiffness, which we define by the condition which maximizes CoM push-off work, generally increases with increasing walking speed, supported by a significant positive correlation between normalized walking speed (Froude number) and selected prosthetic ankle stiffness. This trend agrees with the behavior of the biological ankle-foot complex, which demonstrates an increase in mean stiffness as walking speed increases^28,30,54^. Our results underscore the potential to increase peak power across a range of walking speeds and decrease contralateral limb loading through the use of a microprocessor-controlled variable-stiffness prosthesis, helping to normalize biomechanics of walking and potentially reducing risk factors for knee osteoarthritis development. This result could have important implications for how quasi-passive ankle-foot prostheses are designed, demonstrates the importance of microprocessor-controlled prosthetic ankle stiffness tuning across walking speed, and motivates further development of microprocessor-controlled variable-stiffness systems.

We performed a regression analysis to determine the relationship between prosthesis stiffness and evaluated metrics (Table S2). Peak ankle angle and prosthesis energy return demonstrated a significant linear dependence on joint stiffness across all evaluated walking speeds. Peak prosthesis power showed a significant linear relationship at 1.0, 1.25, and 1.5 m/s, and CoM push-off work at 1.0 and 1.5 m/s. Contralateral limb GRF and knee EAM were not significant to stiffness at any evaluated walking speeds. This regression analysis supports the conclusions of prior investigations on the effect of prosthesis stiffness on biomechanics. As in prior work, we demonstrated that ankle stiffness is linearly and inversely correlated with peak dorsiflexion angle^23,38^ and energy storage and return^23^. Although forefoot stiffness differs from ankle joint stiffness, this work provides a similar result to experiments that analyze the effects of forefoot stiffness changes on dorsiflexion angle, energy return, and prosthetic power^42,44^. The decrease in selected prosthetic stiffness for slower walking speeds is in agreement with initial results regarding user stiffness preference across speeds^23,40^. Interestingly, while energy return and dorsiflexion angle are linearly dependent on stiffness across walking speeds, CoM push-off work and contralateral GRF are not. This result indicates that there are velocity-related differences related to the impacts of ankle stiffness on whole-body biomechanics that a simple minimization of joint stiffness across all gait speeds does not account for.

There is currently not a consensus on specific biomechanical metrics that constitute optimal prosthesis performance. Prior studies have determined optimal device properties based on combined user and prosthetist input^55^, minimization of metabolic cost and contralateral knee loading^56^, or minimization of muscle compensation^57^. This paper quantifies the relationship between prosthesis stiffness and key biomechanical performance metrics: CoM push-off work, prosthetic power, prosthetic energy return, and contralateral limb GRF. We propose that the maximization of push-off power can be considered as a potential clinical metric of prosthesis performance.

Existing commercial ESR prostheses exhibit passive stiffnesses that are higher than the mean stiffness of biological ankle, especially at moderate and slow walking speeds, stairs, and slopes^37^. Prior work has demonstrated a preference for lower-stiffness ESR devices^58^, preference for lower device stiffness at slower walking speeds with a stiffness-adjustable prosthesis^23,40^, and lower stiffness preference from users compared to prosthetists at preferred walking speeds^41^. While it is not clear why ESR prostheses exhibit higher than biological ankle stiffness, we hypothesize that this is influenced by a number of factors such as: bending stress levels that are excessively high for device durability in traditional single beam ESR architectures at biological stiffness levels, desire for increased passive standing stability, accommodating device stiffness desired for fast walking speeds, and prosthetic prescription practices largely based on kinematics rather than kinetics. With the presented multi leaf spring variable-stiffness prosthesis architecture, energy storage and return is increased across walking speeds through stiffness adjustment while maintaining lower peak stress and device durability and allowing for standing stability and stiffness levels corresponding to fast walking. The presented results of device cycle life testing demonstrate the durability of the VSA design and highlight the potential suitability as an everyday use prosthesis. Additionally, cycle life results indicate that any device fatigue effects are unlikely to impact VSA performance during the presented clinical study experiments. Based on the promising results demonstrated in the present study, we believe that stiffness-adjustable prosthetic devices offer benefits beyond passive ESR devices.

Trials were limited to 30 seconds of recorded data for each condition due to the evaluation of 28 distinct experimental conditions. The number of gait cycles included in the gait analysis for each trial condition ranged from 10 to 25, with an average of 19. The Cohen’s d effect size is presented (Table 1) and demonstrates the relative strengths of the observed significant differences in prosthetic joint angle, prosthesis power, energy return, CoM push-off work, and contralateral limb GRF with the VSA compared to the Taleo. For the evaluated metrics, there is a large effect size for prosthetic joint angle, energy return (0.75 m/s and 1.25 m/s), and push-off work (1.0 m/s, 1.25 m/s, and 1.5 m/s). There is a medium effect size for prosthesis power (at 0.75 m/s), energy return (at 1.0 and 1.5 m/s), GRF (at 1.0 and 1.25 m/s), and push-off work (0.75 m/s), and a small effect size for all other significant results. Due to interstride variability, a larger number of gait cycles included in each trial condition would strengthen the work, particularly for the evaluated metrics with a small effect size^59,60^. Prior work has demonstrated a positive correlation between prosthetic energy return and device adaptation time^61^, motivating future experiments to investigate the effects of acclimatization time with the VSA. The majority of experimental conditions were performed with the VSA, which may have inadvertently allowed the participants additional adaptation time for the VSA compared to the Taleo. This effect is anticipated to be minimal because all study participants use ESR prostheses as their daily prosthetic, but warrants further investigation. Additional potential study limitations include the evaluation of walking at 4 discrete treadmill speeds, which may not fully capture the performance of the Taleo at participants’ preferred walking speeds. However, the range of evaluated speeds was selected to encompass typical self-selected walking speeds for people with TTA^62^.

The identified relationship between normalized walking speed (Froude number) and device stiffness that maximizes CoM push-off power has a coefficient of determination of 0.45, indicating that approximately 50% of the variability in selected prosthesis stiffness is not explained by the presented linear model. Additional sources of variation include individual walking patterns, differing rates of acclimatization to an unfamiliar prosthesis, or possible leg-length dependency of ideal prosthesis stiffness. Broader experimentation is needed to identify a comprehensive relationship between walking speed and optimal prosthetic stiffness.

Through this work, we have identified that the prosthetic stiffness that maximizes CoM push-off work for the majority of the study participants at the slower walking speeds of 0.75 m/s, 1.0 m/s, and 1.25 m/s are between 360 Nm/rad and 400 Nm/rad, with the total range of effective mean stiffness of the VSA spanning from 360 Nm/rad to 483 Nm/rad. The VSA target stiffness range was determined based on biological joint mean stiffness values during normal walking^30^, as further described in our prior work^49^, with the stiffness range encompassing the stiffness of the passive ESR device. The prosthesis used for the control condition (Ottobock Taleo) was selected based on the prescribed category according to clinical guidelines based on body mass for each participant^63^. The non-linear torque-angle profiles of the variable stiffness prosthesis and the Ottobock Taleo (Fig. 1E) make a direct comparison of stiffness characteristics difficult, though the mean stiffness for the Taleo was within the evaluated range of the VSA prosthesis for most of the evaluated speeds (1.0 m/s, 1.25 m/s, and 1.5 m/s) (Fig. 1F, Table S1). The stiffness profile of the VSA differs from the Taleo, with stiffness increasing at higher levels of deflections. A more comprehensive analysis of the effects of prosthetic ankle stiffness on CoM push-off power would require a broader range of evaluated stiffnesses as well as more comparable torque-angle profiles between the VSA and passive device. Nonetheless, the present study highlights important findings about the potential to improve CoM push-off power and prosthetic energy storage across walking speed through computer-controllable prosthesis stiffness.

**Table 3:** Study participant information.

Subject	Body mass (kg)	Height (m)	Leg length (m)	Age (y)	t since amp (y)	Sex
1	82.6	1.83	1.00	49	30	Male
2	86.5	1.91	1.05	51	29	Male
3	74.8	1.73	0.88	58	6	Male
4	78.5	1.81	0.92	46	5	Male
5	82.7	1.70	0.84	50	8	Male
6	86.1	1.91	0.98	48	44	Male
7	78.6	1.78	0.94	47	9	Male

Long-term clinical studies are necessary in order to determine the impact of the achieved reduction in contralateral limb loading on health outcomes. Future studies should explore if the results demonstrated in the present study apply to participant groups of varied weight, height, age, and sex, as the current study presents results from study participants who range in body mass from 74.8–86.5 kg, height from 1.70–1.91 m, age from 46–58 years, and all participants were male (Table 3). Ultimately, to provide clinical benefit for prosthesis users of a wide range of body mass and height, the variable stiffness ankle architecture would be scaled such that for each standard prescription class of passive prostheses there is a VSA with a different nominal stiffness range. While the study demonstrated a significant decrease in contralateral limb GRF, a significant decrease in knee EAM was not observed, and further exploration is necessary to determine the potential impact of the variable-stiffness prosthesis on knee EAM through an increase in study population and a reduction in measurement error in the inverse dynamics calculation. In the design of quasi-passive ankle-foot prostheses, we feel microprocessor-controlled variable-stiffness mechanisms are an important area of future research and development.

## Methods

### Experimental protocol

**Figure 5:**
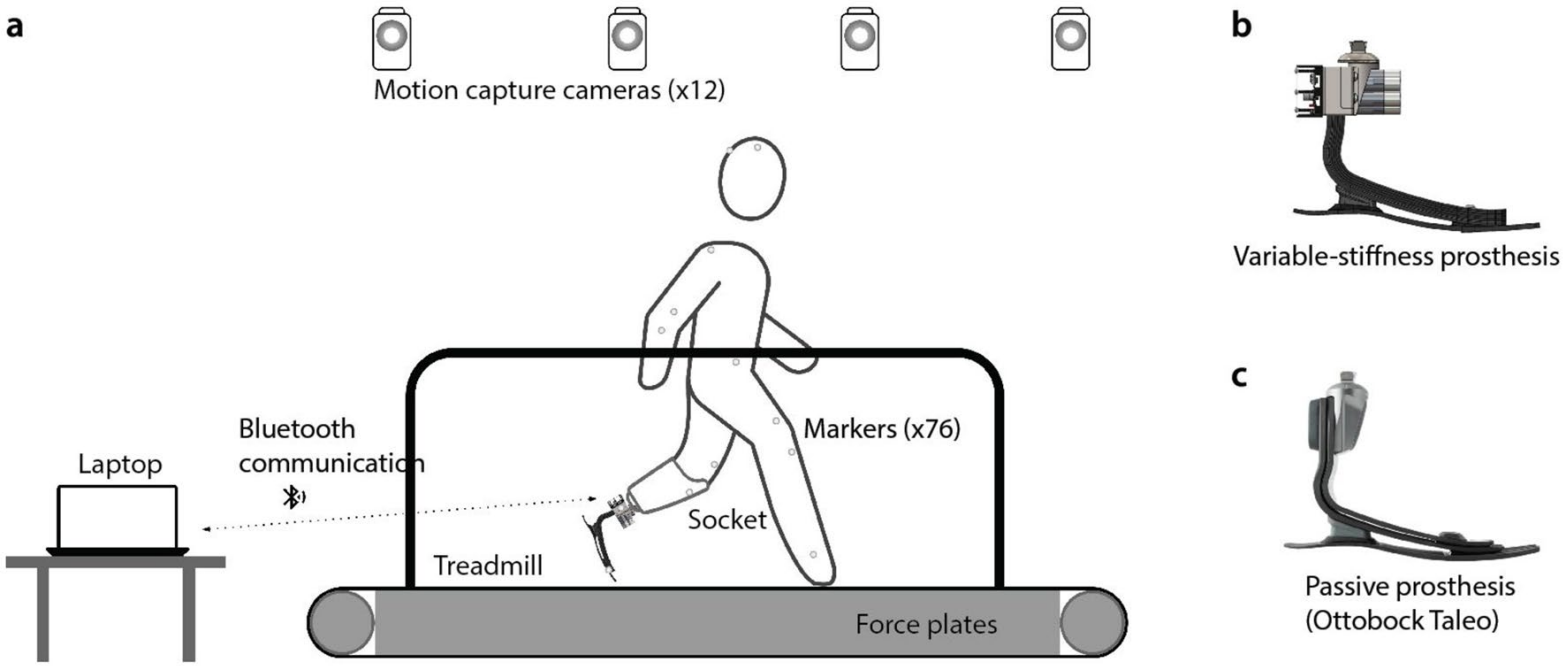
Experimental setup. (**a**) Schematic of experimental setup showing instrumented treadmill, motion capture cameras, motion capture markers, prosthetic socket, and experimental prostheses: variable-stiffness prosthesis (**b**) and passive prosthesis (**c**).

A study was conducted with 7 participants (body mass: $81.5 \pm 4.4$ kg, height: $1.81 \pm 0.08$ m, age: $49.9 \pm 4.0$ years, time since amputation: $18.7 \pm 15.5$ years, sex: male) (Table 3) with unilateral transtibial amputation in order to evaluate the kinetic and kinematic effects of the variable- stiffness prosthesis during walking compared to a passive ESR control foot (Fig. 5). This study was approved by the MIT Committee on the Use of Humans as Experimental Subjects (protocol number: 1609692618, approval date: November 1, 2018) and was performed in accordance with the Declaration of Helsinki. Written informed consent was obtained from all study participants. During the experiment, subjects walked on an instrumented treadmill (FIT, Bertec, Columbus, OH) at the speeds of 0.75 m/s, 1.0 m/s, 1.25 m/s, and 1.5 m/s. Participants walked for 30 seconds for each trial with 30 seconds of rest. A total of 28 trials were performed for each participant. Study conditions included the variable-stiffness prosthesis at 6 distinct stiffness states, as well as a standard passive ESR prosthesis of the subject’s prescribed size and category according to clinical guidelines based on body mass (Taleo 27-5 or 27-6, Ottobock, Duderstadt, Germany)^63^. The torque-angle characteristics of the variable-stiffness prosthesis and the Ottobock Taleo are presented in Fig. 1E, mean stiffness during the trials is shown in Fig. 1F and Table S1. The order of the trials for each stiffness state was randomized. During the trials we recorded motion data with a 12-camera motion capture system (Vero, Vicon Motion Systems, Ltd., Oxford, UK) at a rate of 100 Hz. A custom full-body marker set based on the open source ’3DGaitModelwithSimpleArms’ marker set was used for kinematic data collection, with 5 markers on each foot, 7 on each shank, 7 on each thigh, 4 on the pelvis, 5 on the torso, 4 on each upper arm, 4 on each lower arm, 4 on each hand, and 5 on the head. A split belt treadmill with integrated force plates (FIT, Bertec, Columbus, OH) was used to collect kinetic data (1000 Hz). Data from onboard sensors on the prosthesis (strain gauge, IMU, current sensors) were logged for each trial (500 Hz).

### Variable-stiffness prosthesis

The VSA (Fig. 1A) consists of parallel composite leaf springs that are constrained from sliding relative to a ground spring via computer controlled linear actuators (Fig. 1B). Actuation of the solenoid-driven actuators is controlled by an onboard embedded system. The prosthesis interfaces with a standard prosthetic socket via a pyramid adapter. The same prosthesis design was used for all participants, with left-foot and right-foot versions made to match the side of the amputation. Discrete stiffness control is achieved by selectively constraining sliding of the parallel leaf springs to the ground spring (Fig. 1C). The parallel leaf spring architecture additionally benefits system performance by decreasing peak bending stress in the composite for a given device stiffness, enabling achievement of lower stiffness than is feasible with a single beam architecture. We calculate maximum bending stress for a cantilever beam approximation of the device as in Eq. 3, where M is the peak bending moment, b is spring width, and h is spring thickness. For a given total stiffness and load, the peak stress exhibited in a multi-spring architecture relative to the nominal maximum bending stress in a single beam architecture is given by Eq. 4, where n represents the number of discrete parallel leaf springs. Through this device architecture we can achieve higher spring deflections with lower bending stress than are possible in a single spring design.

3 $$\begin{aligned} \sigma _{max}= & {} \frac{6M}{bh^2} \end{aligned}$$

4 $$\begin{aligned} \sigma _{max,multi}= & {} \root 3 \of {\frac{1}{n}} \sigma _{max} \end{aligned}$$

The highest stiffness state occurs when all five springs are constrained from sliding (Fig. 1C-i), the lowest stiffness state occurs when all springs are unconstrained and able to freely slide (Fig. 1C-iii), and intermediate stiffness states occur with any combination of constrained springs (Fig. 1C-ii). The prosthesis has 32 discrete stiffness states. For this study, 6 stiffness settings were used with nominal prosthetic stiffness ranging from 375–569 Nm/rad (Fig. 1E,F). Prosthetic stiffness was characterized for the VSA and Taleo through benchtop testing on a material testing system (Model 5969 Material Testing System, Instron, Norwood, MA) through the use of a custom test fixture, as described in^49^. To expand on previously presented stiffness characterization^49^, the evaluation was conducted for the 6 selected stiffness states of the VSA with a maximum load of 1400 N applied to the prosthesis at a rate of 50 N/s, while linear deflection at the point of load application was measured (Model 5969 Material Testing System, Instron, Norwood, MA) and angular deflection of the prosthetic toe was measured (AXISENSE-2 USB90, TE Connectivity, Schaffhausen, Switzerland). Each stiffness state was measured 3 times and the mean stiffness is calculated. The variable-stiffness prosthesis exhibits a non-linear stiffness with device stiffness increasing with increasing deflection angle (Fig. 1E). The durability of the VSA was evaluated experimentally to determine if fatigue effects are likely to impact device performance over the duration of the clinical study. Cycle life testing was performed (Ottobock HealthCare, Salt Lake City, UT) with the variable-stiffness prosthesis in the lowest-stiffness configuration to ISO 22675 standards. Reference ankle moment was measured at the ISO reference ankle (08 mm above ground plane) during loading on a 15 degree heel plate and 20 degree toe plate. More details of device design and functionality are presented in^49^.

### Study design

The objective of the present study was to evaluate the hypothesis that a variable-stiffness prosthesis will demonstrate an increase in energy return and push-off power across walking speeds compared to a passive device. Additionally, we hypothesized that the stiffness that provides the largest improvement to whole-body biomechanics will decrease as walking speed decreases. To evaluate this hypothesis, we implemented a repeated measures (within subjects) study design. Participant inclusion criteria include an all-timestep RMS error in the inverse kinematics analysis of less than 0.04 for all marker positions, based on best practice recommendations^64^. One participant was excluded from the analysis due to high marker errors caused by marker movement during the trial. Gait cycles were excluded from analysis if the unified deformable ankle power or EAM were greater than 2 standard deviations from the median, in order to identify gait cycles during which foot contact occurred on both force plates, excessive marker motion was present, or gait cycles were segmented incorrectly. On average, 19.1 gait cycles (standard deviation: 3.4) were included in analysis for each trial, with a range from 10 to 25 gait cycles. On average 4.6 (standard deviation: 2.6) gait cycles were removed from analysis for each condition.

### Data analysis

The data were segmented into individual gait cycles for each trial via a GRF-based heel strike detection algorithm with a force threshold of 50 N (Vicon Nexus), and verified with manual visual inspection. Data were processed using OpenSim (OpenSim 4.3, Simbios, Stanford, CA) and AddBiomechanics^64,65^. A base subject model with unilateral TTA was created using mass and inertial properties of the lower leg on the prosthesis side based on standard residual limb dimensions and properties^66^. For each evaluated condition, mass of the lower-leg segment was adjusted to reflect the appropriate mass of the variable-stiffness device or the ESR device. AddBiomechanics^65^ was used to optimally scale the model for each subject based on marker positions^65^. Inverse kinematic calculations were also performed using AddBiomechanics^65^. Inverse dynamics were performed in OpenSim (OpenSim 4.3, Simbios, Stanford, CA)^64^. A 3rd order zero-lag low-pass Butterworth filter was used to filter kinematic (6 Hz) and kinetic (12 Hz) data using Matlab (MATLAB R2021a, MathWorks, Natick, MA, USA). Outlier gait cycles were excluded as described in the above Study Design section with (*rmoutliers*, MATLAB R2021a, MathWorks, Natick, MA, USA). The selected stiffness state for each subject and each speed was defined as the VSA stiffness that maximized mean CoM push-off work.

### Kinematic and kinetic analysis

Prosthetic-side joint angles were calculated using inverse kinematics (through AddBiomechanics^65^) as described in the Data Analysis section.

Prosthetic ankle power was calculated using a unified deformable model due to the tendency of inverse dynamics calculations to inaccurately quantify joint power for a deformable prosthesis without a fixed ankle joint^67,68^. Prosthetic power is calculated as in Eq. 5, where $F_{grf}$ is the ground reaction force, $M_{grf}$ is the ground reaction moment, $\omega _{s}$ is the angular velocity of the shank, and $v_{sdef}$ is the deformation velocity of the shank. Equation 6 calculates $v_{sdef}$ , where $v_{sCOM}$ is the CoM velocity of the shank segment and $r_{com-cop}$ is the radius from the CoM of the shank segment to the center of pressure.

5 $$\begin{aligned} P_{prosthesis}= & {} F_{grf}\cdot v_{sdef} + M_{grf}\cdot \omega _{s} \end{aligned}$$

6 $$\begin{aligned} v_{sdef}= & {} v_{sCOM} + \omega _{s} \times r_{com-cop} \end{aligned}$$

Prosthesis energy was calculated by integrating prosthetic ankle power across time (*cumtrapz*, MATLAB) for the duration of the gait cycle. Energy return is calculated as the difference in energy from the time where peak negative power occurs during late stance to toe-off.

CoM power on the prosthetic side was determined by taking the dot product of ground reaction force from the prosthetic-side leg ($F_{grfprosthesis}$) and CoM velocity ($v_{com}$). CoM push-off work was calculated as the time integral (*cumtrapz*, MATLAB) of CoM power for the trailing leg (prosthetic side) during the step transition (Eq. 7). The time integral was taken from the time of the maximum negative power during late stance ($t_1$) to the time of the peak power during push-off ($t_2$). CoM power was determined by taking the dot product of ground reaction force ($F_{grfprosthesis}$) and CoM velocity ($v_{com}$).

7 $$\begin{aligned} W_{push-off}=\int _{t_1}^{t_2}P_{push-off} =\int _{t_1}^{t_2}F_{grfprosthesis} \cdot v_{com} \end{aligned}$$

CoM power on the contralateral side was determined by taking the dot product of ground reaction force ($F_{grfleading}$) and CoM velocity ($v_{com}$). CoM collision work was calculated as the time integral of CoM power (*cumtrapz*, MATLAB) for the leading leg during collision during the step transition (Eq. 8). The time integral was taken from heel strike ($t_3$) to the time where the maximum negative power occurs ($t_4$).

8 $$\begin{aligned} W_{collision}=\int _{t_3}^{t_4}P_{collision} = \int _{t_3}^{t_4}F_{grfleading} \cdot v_{com} \end{aligned}$$

External adduction moment of the contralateral limb was calculated through inverse dynamics (OpenSim 4.3, Simbios, Stanford, CA)^64^. The internal OpenSim inverse dynamics tool was used to calculate joint torques.

### Selected prosthetic stiffness

We used the Froude number to compare relative walking speeds between subjects of different heights and leg lengths. The Froude number is a dimensionless number that represents the ratio of centripetal force to gravitational force in an inverted pendulum model of walking^69^. The Froude number scales proportionally with walking speed and inversely with leg length, with the walk to run transition typically occurring at a value of 0.5^69^. The Froude number was calculated as in Eq. 9, where *v* is walking velocity ($\frac{m}{s}$), *g* is the acceleration due to gravity ($\frac{m}{s^2}$), and $l_{leg}$ is leg length (*m*) from the ground to the greater trochanter of the femoral head^69^. A first-order linear regression (*fitlm*, MATLAB) was performed to determine the relationship between Froude number and prosthetic stiffness. The correlation coefficient (*corrcoef*, MATLAB) and 95% confidence interval of the linear regression model (*predict*, MATLAB) were calculated.

9 $$\begin{aligned} Fr=v^2/gl_{leg} \end{aligned}$$

To analyze prosthesis stiffness across walking speeds, we calculate the mean stiffness of the variable-stiffness prosthesis for each subject at each walking speed across the entire stance phase, based on the peak ankle torque calculated from the inverse dynamics model, and the measured device stiffness profile from benchtop testing (Eq. 10). The stiffness for each prosthesis setting at each walking speed is shown in Fig. 1F and Table S1.

10 $$\begin{aligned} K=\frac{\Delta \tau }{\Delta \theta } \end{aligned}$$

### Statistical methods

A Shapiro–Wilk test (*swtest*, MATLAB) was first performed on all paired results to test for normality of the differences between pairs. For all conditions except peak joint angle at 1.0 m/s the Shapiro–Wilk test did not reject the null hypothesis, indicating a normal distribution. All conditions that met assumptions of normality were analyzed with a paired t-test (*ttest*, MATLAB) with a significance level of 0.05. The peak joint angle at 1.0 m/s condition was evaluated with a paired Wilcoxon signed rank test due to non-normality (*signrank*, MATLAB). Effect size for all conditions was computed using a Cohen’s d test with a confidence level of 0.05 (*meanEffectSize*, MATLAB). All statistical analysis was performed in MATLAB (MATLAB R2022a, MathWorks, Natick, MA, USA). Uncertainty measurements are presented as $\pm 1$ standard deviation in all plot error bars or shaded regions.

### Supplementary Information

Supplementary Information 1.

Supplementary Information 2.

## Data Availability

All data needed to evaluate the conclusions of the paper are available in the paper or the Supplementary Information. The original dataset is included in the Supplementary Information.
